# Retroperitoneal Abscess: A Rare Localization of Tubercular Infection

**DOI:** 10.1155/2010/475130

**Published:** 2010-10-31

**Authors:** Sami Karoui, Norsaf Bibani, Afef Ouaz, Meriem Serghini, Faouzi Chebbi, Kais Nouira, Ines Chelly, Jalel Boubaker, Zoubeir Ben Safta, Emna Menif, Moncef Zitouna, Azza Filali

**Affiliations:** ^1^Department of Gastroenterology A, La Rabta Hospital, Tunis, 1007, Tunisia; ^2^Department of Surgery, La Rabta Hospital, Tunis, 1007, Tunisia; ^3^Department of Radiology, La Rabta Hospital, Tunis, 1007, Tunisia; ^4^Department of Pathology, La Rabta Hospital, Tunis, 1007, Tunisia

## Abstract

Incidence of tuberculosis infection has considerably increased during the past 20 years due to the HIV pandemic and continues to be one of the most prevalent and deadly infections worldwide. Extrapulmonary tuberculosis lacks specific clinical manifestation and can mimic many diseases. It can invade neighbouring tissue and form a big cyst with manifesting clinical symptoms. We describe a rare case of 31-year-old immunocompetent man affected by a retroperitoneal abscess secondary to tubercular infection. Exploratory laparotomy and histopathological examinations of tissue were required for achieving diagnosis of tuberculosis. No pulmonary or spinal involvement was identified. The patient was successfully treated with standard four-drug antitubercular therapy.

## 1. Introduction

Despite increasing awareness and availability of better imaging and other diagnostic tests, extrapulmonary tuberculosis remains a difficult diagnosis to make, due to its often nonspecific and protean manifestations. However, as there are no pathognomonic imaging findings, the diagnosis ultimately rests on histopathological and microbiological confirmation. Intestine, peritoneum, and lymph nodes are the more common localizations when tuberculosis affects the abdomen [1].

We report a rare case of massive retroperitoneal abscess in which the tubercular aetiology was established after histopathological exam with favourable evolution after antitubercular medications.

## 2. Case Report

 A 31-year-old man was admitted to our department in April 2009 with the history of right upper abdominal pain, fever, and bilious vomiting. In his medical history, there was a posttraumatic splenic hematoma in August 2007 which required splenectomy. No tuberculosis exposure was mentioned. Physical examination revealed a body temperature to 38° and a painful mass over the right flank region. Laboratory results revealed an increased erythrocyte sedimentation rate (120 mm/1°) and C-reactive protein (80 mg/L) associated with leucocytosis (19000/mm3, 83% neutrophils, and 7% lymphocytes), mild anaemia (haemoglobin 11,2 g/dL), and thrombocytosis (platelets = 773000/UL). Liver function tests, blood urea nitrogen, and serum creatinin were within normal limits. A chest film was normal. 

**Figure 1:**
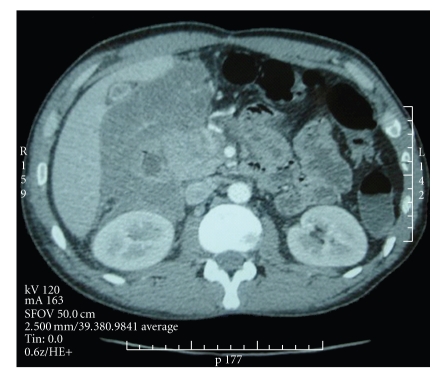
CT-scan showing the retroperitoneal mass.

An abdominal computed tomography revealed a large cystic retroperitoneal mass (184 × 126 mm) extending from the flank to right iliac fossa, pushing the right colon anteriorly, surrounding the anterior sides of the abdominal aorta and the inferior vena cava. It is located behind duodenum, ahead the kidney and under the liver, without signs of invasion and without lymph node involvement. The lesion had spontaneous density between 12 and 30 Hounsfield units. There was a complete enhancement of the lesion (Figure 1).

**Figure 2:**
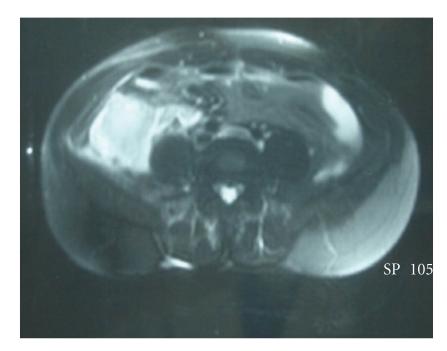
MR characteristics of the retroperitoneal mass.

An MR scan revealed a cystic mass, hypointense in T1, and hyperintense in T2 with an enhancement of the entire lesion (Figure 2).

Percutaneous aspiration of the mass, under sonographic guidance, yielded mucinous fluid but insufficient for mycobacteriological examination. Blood cultures for aerobic and anaerobic bacteria did not produce any growth. 

Thus, a laparotomy was performed. It revealed a large retroperitoneal yellowish mass containing pus. It is extending from the root of the mesentery to the vena cava. The access to this mass is difficult, and it required dividing the coloepiploic attachments. There were no carcinosis or ascits. The small bowel and the appendix were normal. 

**Figure 3:**
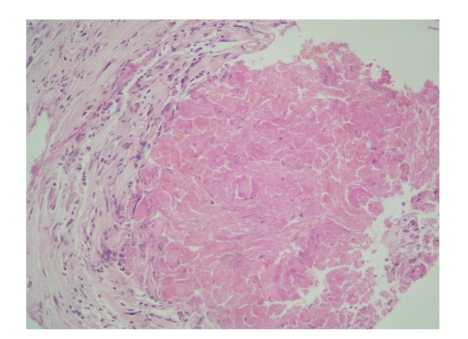
Histological examinationscore (HES) showing granulomatous inflammation with caseous material.

The histopathological examination showed epitheloid granulomas with necrosis, giant cells, and lymphoepitheloid infiltrate (Figure 3). Culture and polymerase chain reaction of tuberculosis using the surgical specimen were not performed. The diagnosis of retroperitoneal tuberculosis was established. There were not other tuberculosis involvements: computed tomography chest was normal, and MR scan of the spine excluded a spondylodiscitis. HIV test was negative.

Antitubercular therapy was started with isoniazid (300 mg once daily), rifampicin (600 mg once daily), pyrazinamide (2000 mg once daily) and ethambutol (1200 mg once daily), and was given for 9 months. No others antibiotics were administrated. The cyst gradually subsided and disappeared with a normal abdominal computed tomography 6 and 12 months later.

## 3. Discussion

Retroperitoneal abscesses are often polymicrobial, and the predominant isolates are Escherichia coli, klebsielle pneumoniae, enterococcus spp., and staphylococcus aureus [2]. It may complicate perforated colonic carcinoma, crohn's disease of the bowel, diverticulitis, perforated appendicitis, or trauma [1, 3–6]. Other clinical conditions associated with the formation of retroperitoneal abscess include pyelonephritis, renal carbuncle, tuberculosis, trauma, and cancer [1]. Abdominal tuberculosis is uncommon and generally found in patients with severe disseminated disease [7]. Most of cases resulted from a miliary tuberculosis which is usually secondary to lympho-haematogenous spread from a primary lung focus, or may result from a direct extension from an adjacent organ or bacilli ingestion [8]. However, tuberculosis more frequently affects nonimmunocompetent hosts, such as HIV-infected subjects or patients with diabetes mellitus and end-stage renal disease [9]. 

In our patient, retroperitoneal location of tuberculosis was unique. There is no lung or spine involvement associated, such as chest scan and spinal MRI were normal. Immunosuppression was excluded given the past medical history, negative routine blood tests, normal immunoglobulin levels, and negative HIV tests. Other bacterial infection was not found in blood culture, and the percutaneous aspiration of the fluid was insufficient for mycobacteriological examination. 

Our country is considered as an endemic area, thus, reactivation of latent infection should be regarded as possible, even in an immunocompent host. 

Delayed presentation and large abscess formations, like in our patient, in cases of tuberculosis infections occur more frequently rather than in cases of pyogenic infections.

The various symptoms were shown on tubercular infection; on the other side most patients has latent infection. In the few cases reported, the diagnosis of retroperitoneal tuberculosis infection was not confirmed by only the interview [10–13]. The tuberculin test was performed and was positive in 2 cases [10, 12] and the QuantiFERON-TB interferon-gamma (QFT) was performed and was positive in one case [10]. 

Tuberculosis may be detected by chest X-ray (33%) and abdominal computed tomography (88%), which are the most frequently used imaging modalities but there were not pathogenomic criteria [14, 15]. Thus, there are not specific imaging techniques for retroperitoneal abscess secondary to tubercular infection.

 The polymerase chain reaction is rapid and reliable test, and the results are available within 6.5 hours, even for contaminated specimens, with reasonable sensitivity (76,4%) and excellent specificity (99,8%) [15]. Tissue and body fluid specimens have not been validated as reliable in large case series [16, 17].

The use of fine needle aspiration in the diagnosis of retroperitoneal tuberculosis has been evaluated by several studies. This method can be the best procedure to procure tissue sample for diagnosis and can reduce the number of laparotomies performed for infectious and nonresectable malignant retroperitoneal disease [18]. 

 However, in the few cases reported about retroperitoneal abscess with tubercular infection, the diagnostic was usually confirmed by surgery [10–13]. The pathology study of the specimens revealed granuloma and giant cells with necrotic tissue. The culture of the specimens was performed and was positive in 2 cases [10, 13]. 

Standard four-drug antitubercular therapy is the mainstay in the management of abdominal tuberculosis for at least 6 months [14].

## 4. Conclusion

Tuberculosis is still common in our country. Tubercular aetiology of an abdominal abscess must be suspected in immunocompetent hosts, even in the presence of isolated extrapulmonary localization.
